# Comparative transcriptomics of human multipotent stem cells during adipogenesis and osteoblastogenesis

**DOI:** 10.1186/1471-2164-9-340

**Published:** 2008-07-17

**Authors:** Marcel Scheideler, Christian Elabd, Laure-Emmanuelle Zaragosi, Chiara Chiellini, Hubert Hackl, Fatima Sanchez-Cabo, Sunaina Yadav, Kalina Duszka, Gerald Friedl, Christine Papak, Andreas Prokesch, Reinhard Windhager, Gerard Ailhaud, Christian Dani, Ez-Zoubir Amri, Zlatko Trajanoski

**Affiliations:** 1 Institute for Genomics and Bioinformatics and Christian Doppler Laboratory for Genomics and Bioinformatics, Graz University of Technology, Petersgasse 14, 8010 Graz, Austria; 2 ISBDC, Université de Nice Sophia-Antipolis, CNRS, 28 avenue de Valrose, 06100 Nice, France; 3 Genomics Unit, Centro Nacional de Investiganciones Cardiovasculares, Madrid, Spain; 4 Department of Orthopaedics, Medical University of Graz, Graz, Austria

## Abstract

**Background:**

A reciprocal relationship between bone and fat development in osteoporosis is clinically well established. Some of the key molecular regulators involved in this tissue replacement process have been identified. The detailed mechanisms governing the differentiation of mesenchymal stem cells (MSC) – the key cells involved – are however only now beginning to emerge. In an attempt to address the regulation of the adipocyte/osteoblast balance at the level of gene transcription in a comprehensive and unbiased manner, we performed a large-scale gene expression profiling study using a unique cellular model, human multipotent adipose tissue-derived stem cells (hMADS).

**Results:**

The analysis of 1606 genes that were found to be differentially expressed between adipogenesis and osteoblastogenesis revealed gene repression to be most prevalent prior to commitment in both lineages. Computational analyses suggested that this gene repression is mediated by miRNAs. The transcriptional activation of lineage-specific molecular processes in both cases occurred predominantly after commitment. Analyses of the gene expression data and promoter sequences produced a set of 65 genes that are candidates for genes involved in the process of adipocyte/osteoblast commitment. Four of these genes were studied in more detail: *LXRα *and phospholipid transfer protein (*PLTP*) for adipogenesis, the nuclear receptor *COUP-TF1 *and one uncharacterized gene, *TMEM135 *for osteoblastogenesis. *PLTP *was secreted during both early and late time points of hMADS adipocyte differentiation. *LXRα*, *COUP-TF1*, and the transmembrane protein *TMEM135 *were studied in primary cultures of differentiating bone marrow stromal cells from healthy donors and were found to be transcriptionally activated in the corresponding lineages.

**Conclusion:**

Our results reveal gene repression as a predominant early mechanism before final cell commitment. We were moreover able to identify 65 genes as candidates for genes controlling the adipocyte/osteoblast balance and to further evaluate four of these. Additional studies will explore the precise role of these candidate genes in regulating the adipogenesis/osteoblastogenesis switch.

## Background

That the decrease in bone volume associated with osteoporosis is accompanied by an increase in marrow adipose tissue is clinically well known [1]. Pharmacological inhibition of this tissue replacement process could provide a novel mode of treatment for this disorder. A rational approach to drug development requires knowledge of the underlying molecular mechanisms. For both adipogenesis and osteoblastogenesis, many key regulators have been identified using established cell model systems. The detailed mechanisms that control the differentiation of mesenchymal stem cells (MSC) – the key cell type involved – are however only beginning to emerge.

Adipogenesis is a highly regulated process in which a coordinated cascade of transcription factors leads to the formation of mature adipocytes [2,3]. This cascade begins with the transient expression of CCAAT/enhancer binding protein β (*C/EBPβ*) and *C/EBPδ *which activate *C/EBPα *and peroxisome proliferator-activated receptor γ (*PPARγ*). *C/EBPα *and *PPARγ *together coordinate the expression of adipogenic genes underlying the phenotype of terminally differentiated adipocytes. This terminal differentiation is characterised by the induction of genes including glycerol-3-phosphate dehydrogenase (*GPDH*), hormone-sensitive lipase (*HSL*), fatty acid synthase (*FASN*), fatty acid binding proteins (*FABPs*), perilipin (*PLIN*), and the production and secretion of adipokines such as leptin (*LEP*), adiponectin (*ADIPOQ*), adipsin (*CFD*), tumor necrosis factor alpha (*TNFα*), visfatin (*NAMPT*) and retinol binding protein 4 (*RBP4*). Additional transcription factors, such as sterol-regulatory element binding transcription factor 1 (*ADD1/SREBP1*) can further modulate this terminal differentiation process [3].

Osteoblastogenesis is also a highly coordinated process and is initiated by the transcription factors runt-related transcription factor 2 (*RUNX2*) and osterix (*OSX*), whose expression is regulated by β-catenin, the homeobox protein *MSX2*, and a transcriptional coactivator with PDZ-binding motif (*TAZ*) coactivating *CBFA1 *and repressing *PPARγ *[4,5]. Bone morphogenetic proteins (*BMP*s) promote bone formation by stimulating the proliferation and differentiation of osteoblasts [6]. *BMP*s elicit their cellular effects via specific type I and II serine/threonine receptors [7]. This cascade leads to the terminal osteoblast phenotype that is characterised by calcification of the extracellular matrix (ECM). The genes involved in this mineralization process include noggin (*NOG*), osteonectin (*SPARC*), osteoprotegerin (*OPG*), collagens *COL1A1 *and *COL1A2*, matrix Gla protein (*MGP*), matrilin-3 (*MATN*), and estrogen receptor 1 (*ESR1*) which are differentially expressed in the developing human bone [8-14].

Despite intensive research efforts focusing on the individual differentiation pathways, little is known about the molecular mechanisms that drive final lineage commitment. The small number of candidate genes identified to date include *MSX2 *and *C/EBPβ *which are involved in the reciprocal switch between adipocyte and osteoblast differentiation [15,16], and *FKBP5 *which is up-regulated in a differentiation-independent manner in mesenchymal lineages [17]. These genes were identified either using established cell lines or bone marrow and adipose tissue-derived multipotent MSCs able to differentiate into multiple cell lineages including chondrocytes, osteoblasts and adipocytes [18,19]. Both types of model systems have associated advantages and disadvantages. Cell lines for instance represent a single genetic background but are karyotypically heterogeneous. Primary cells on the other hand have normal karyotypes but are genetically heterogeneous since they are derived from multiple donors.

In an attempt to address the transcriptional regulation of the adipocyte/osteoblast balance in a comprehensive and unbiased manner we have applied large-scale gene expression profiling to a human multipotent adipose-derived stem (hMADS) cell-derived cell line that exhibits a normal karyotype, high self-renewal capacity and an ability to differentiate into different cell types including adipocytes and osteoblasts and to support *in vivo *regenerative processes [20-23]. We asked the question if the global expression profiles were different between the stages before and after lineage commitment. In a first step we performed a detailed characterization of the model and then used microarrays and in-depth bioinformatics analyses to comprehensively study gene expression changes associated with osteoblastogenic and adipogenic differentiation. For both lineages genes and biological processes were found to be predominantly down-regulated prior to commitment. We were able to provide computational evidence that this repression is mediated by miRNA. We further identified novel candidate genes involved in the switch between adipocyte and osteoblast differentiation and confirmed their lineage-dependent transcriptional activation in human primary cells.

## Results

### hMADS cells represent a unique model for the study of adipogenesis and osteoblastogenesis

To characterize the hMADS cell model, quantitative RT-PCR assays were performed for 33 selected genes using RNA harvested at the reference time point and at the time points 5, 7, and 8 for both differentiation pathways (materials and methods).

**Figure 1:**
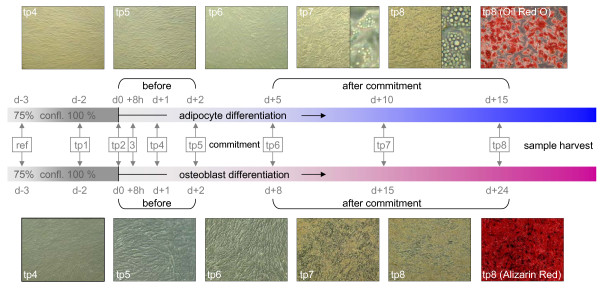
**Adipocyte and osteoblast differentiation of hMADS cells.** Time points for reference and sample harvesting are indicated. Differentiation in both lineages was monitored including oil red O staining of lipid droplets in terminal adipocyte differentiation and Alizarin red staining of calcium deposition in the extracellular matrix in terminal osteoblast differentiation.

Adipocyte differentiation of hMADS cells resulted in an altered cell morphology as shown in Figure 1. Central players of the adipogenesis-specific transcriptional network including *C/EBPβ*, *C/EBPδ*, *PPARγ2*, *C/EBPα*, and *SREBP1c *were found to be regulated in a differentiation-dependent manner (see Additional file 1). In addition, genes characteristic of the terminally differentiated phenotype were found to be up-regulated. These included *GPDH*, *HSL*, *FABP4*, *FABP5*, *PLIN*, *FASN *(genes involved in lipid metabolism), *FXR *(a regulator of cholesterol homeostasis), *GLUT4 *(associated with systemic glucose homeostasis), *PEPCK *(associated with adipocyte glyceroneogenesis) and the genes encoding adipokines *LEP*, *ADIPOQ*, CFD, *TNFα*, *NAMPT*, and *RBP4 *(see Additional file 1).

Osteoblastic differentiation of hMADS cells resulted in calcium deposition in the extracellular matrix (Figure 1). Transcriptional activators known to be expressed in the osteogenic lineage including *CBFA1 *and *MSX2 *were consistently up-regulated during osteoblast differentiation. *BMP4 *and *BMP6*, members of the protein family that induce bone formation at extracellular sites, and the BMP receptors *BMPR1B *and *BMPR2 *were induced. Genes involved in the development and homeostasis of the calcification of the ECM including *NOG*, *SPARC*, *OPG*, *COL1A1*, *COL1A2*, *MGP*, *MATN3*, and *ESR1 *(see Additional file 1) were also found to be up-regulated.

Hence, the normal karyotype, evidence for transcriptional activation of the master regulators, and the biochemical confirmation of the differentiated phenotypes, confirm hMADS cells as a powerful model for the study of the adipocyte/osteoblast balance.

### Lineage-specific molecular processes are predominantly activated after commitment

**Figure 2:**
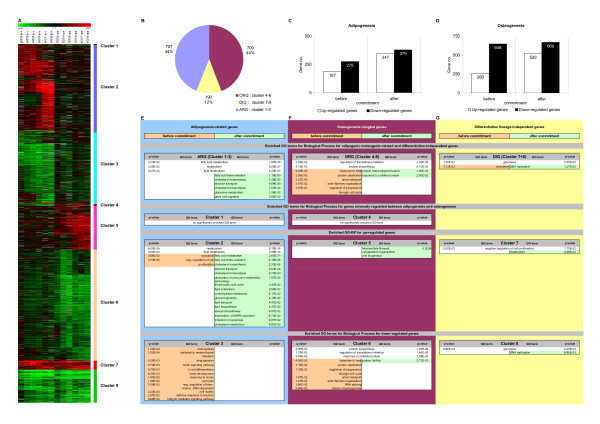
**Differentially expressed genes and GO term analysis**. A) Lineage-specific gene clustering. Shown is a clustering of 1,606 genes to be more than twofold up- or down-regulated in at least one time point after differentiation induction. Genes were grouped into six fate-specific clusters, three with distinct profiles specific for adipogenesis, three with distinct profiles for osteoblastogenesis, and two fate-independent clusters with common profiles in both differentiation lineages. Relative expression levels (log_2 _ratios) for genes at different time points are shown and color coded according to the legend at the top (left) and expression profile (mean ± standard deviation) for each cluster (right). B) Distribution of differentially expressed genes for the three main clusters with adipogenesis-related (ARG), osteoblastogenesis-related (ORG), and differentiation lineage-independent (DIG) profiles. C)-D) Numbers of up-regulated and downregulated genes before and after commitment in both lineages. E) Distribution of significantly enriched GO terms for genes with adipogenic-related profiles (ARG: cluster 1–3) before and after commitment. F) Distribution of significantly enriched GO terms for genes with osteogenic-related profiles (ORG: cluster 4–6) before and after commitment. G) Distribution of significantly enriched GO terms for genes with differentiation-independent profile (DIG: cluster 7+8) before and after commitment.

Expression profiles were generated during differentiation in both lineages and the expression data validated using RT-PCR (see Additional file 2). 41 genes indicative for the adipogenic lineage, 22 genes indicative for the osteogenic lineage, and 37 genes potentially involved in self-renewal with common expression profiles were studied. A high degree of correlation was found (r^2 ^= 0.81), similar to a previous study [24], thus confirming the validity of the microarray data. An overview of the expression profiles of the differentiating cells and GO term analysis are presented in Figure 2A. A subset of genes which had at least 13 out of 16 present expression values (eight time points per differentiation experiment) for both differentiation studies were chosen for further analysis. Out of this subset, a total of 1606 genes could be identified as significantly differentially expressed after induction of differentiation (see Methods section, Microarray data analysis; see Additional file 2). These genes were clustered according to adipogenic-related (ARG), osteogenic-related (ORG), and differentiation-independent (DIG) profiles in both lineages (Figure 2A). Our gene expression data demonstrated an equal percentage of genes to be specifically regulated during adipocyte and osteoblast differentiation (44%), with 12% of genes sharing a common profile in both differentiation lineages (Figure 2B). A large proportion of all 1606 differentially expressed genes were found to be down-regulated (Figure 2C–D, see Additional file 3).

hMADS cells have the ability to differentiate into adipocytes or osteoblasts. After induction towards one of these lineages, they retain the ability to be committed to the other one by changing the differentiation medium and cocktail for a limited time. A medium and cocktail change at or later than time point 6 did not switch the differentiation pathways indicating that the final and irreversible commitment to a given lineage takes place between time points 5 and 6 (data not shown). We therefore analyzed the expression profiles between the early stages of differentiation prior to commitment (time points 3 to 5) and at the late stages of differentiation after commitment (time points 6 to 8). Genes in each cluster were sub-categorized according to their differential expression before and/or after commitment.

In order to characterize the lineage-specific and lineage-independent gene expression clusters, we categorized the genes in each cluster with available RefSeq annotation and Gene Ontology (GO) terms and extracted the GO terms for biological processes which are significantly over-represented (Figure 2E–G). Surprisingly, of the large number of genes found to be differentially expressed during both differentiation pathways, only a small number of significantly enriched GO terms were found before commitment. As expected, ARG are involved in *metabolism*, *fatty acid and lipid metabolism*, *fatty acid beta-oxidation*, and *cholesterol biosynthesis and homeostasis*.

Thus, although a large number of genes were found to be differentially expressed during differentiation, lineage-specific molecular processes were transcriptionally up-regulated only after commitment.

### Predominant gene repression before commitment in adipocyte and osteoblast differentiation pathways

Genes involved in the *hematopoietic system*, *angiogenesis*, *B-cell differentiation *and *epithelial to mesenchymal transition *were repressed during adipocyte differentiation, (cluster 3). Interestingly, genes involved in *notch signaling*, whose down-regulation is commonly considered to be a prerequisite for the adipogenic pathway, were significantly enriched [1]. This gene repression appeared exclusively before commitment to the adipogenic lineage.

During osteoblast differentiation, the GO terms *protein biosynthesis*, *regulation of translational initiation*, and *response to unfolded protein *were enriched by genes repressed before and after commitment (cluster 6). Interestingly, only *protein folding *was found to be down-regulated after commitment, whereas *response to heat*, *protein catabolism*, *regulation through cell cycle*, *anion transport*, *actin filament organization*, *RNA splicing *and *cellular morphogenesis *were repressed before commitment to the osteogenic lineage.*Glycolysis *was statistically over-represented for genes down-regulated before and after commitment, whereas *DNA replication *only after commitment (cluster 8).

Taken together, the results show that the majority of genes were down-regulated, with this down-regulation occurring both before and after commitment. Down-regulated genes were predominant prior to commitment for genes in all of the three main categories, ARG, ORG, and DIG. On the other hand, the number of up-regulated genes doubled from early to late differentiation stages.

### Computational analyses reveal that miRNA targeting corresponds with prevalent down-regulation of genes before commitment

We have recently shown that during adipogenesis a large number of mRNAs might be potential targets for microRNAs (miRNAs) [24]. miRNAs are an abundant class of endogenous, small non-coding RNAs (19–25 nucleotides) that negatively regulate gene expression at the post-transcriptional level by base pairing with the 3'-untranslated region (3'-UTR) of target messenger RNAs. Several studies have demonstrated the involvement of miRNAs in gene regulation, metabolism, cell differentiation, and development [25-30]. Well over one third of mammalian genes appear to be conserved miRNA targets [31]. Rules for target recognition, for instance the seven-nucleotide (7-nt) miRNA seed sequence, defined as positions 2–8 at the 5' end of the miRNA, combined with features of site context, were applied to predict and discover human miRNA targets [31,32].

We performed a miRNA binding site analysis to identify miRNAs potentially targeting genes regulated during adipocyte or osteoblast differentiation and significantly over-represented miRNA targets in the eight distinct gene clusters. Out of the 1606 genes identified as being differentially expressed during differentiation the 3'-UTR sequence could be obtained for 1147 of them. All of these had at least one exact antisense match with the 7-nt seed (base 2–8 at the 5' end) from the 470 human miRNA sequences (19–25 base pairs). 915 genes (79.8%) had at least one match for significantly over-represented miRNAs whose 7-nt seed exactly matched only to 16844 genes (71.3%) among the entire 23.611 3'-UTR sequence set. The distribution of statistically enriched 3'-UTR miRNA motifs varied across the clusters, with genes in cluster 1 having no detectable motifs and genes in cluster 5 having the most miRNA motifs. In summary, 80% of all genes with a unique 3'-UTR that were differentially expressed during adipocyte and osteoblast differentiation are potential targets for 30 miRNAs with significantly over-represented motifs (see Additional file 5).

We performed the analysis of significantly over-represented miRNA binding sites for genes regulated before and after commitment for both differentiation pathways. Within all clusters, we found that the ratio of the number of genes containing a miRNA seed match in the 3'-UTR before and after commitment was comparable with the ratio for all genes regulated before and after commitment in a cluster (see Additional file 4).

### Novel candidate genes for the lineage commitment revealed by gene expression and promoter analyses

**Figure 3:**
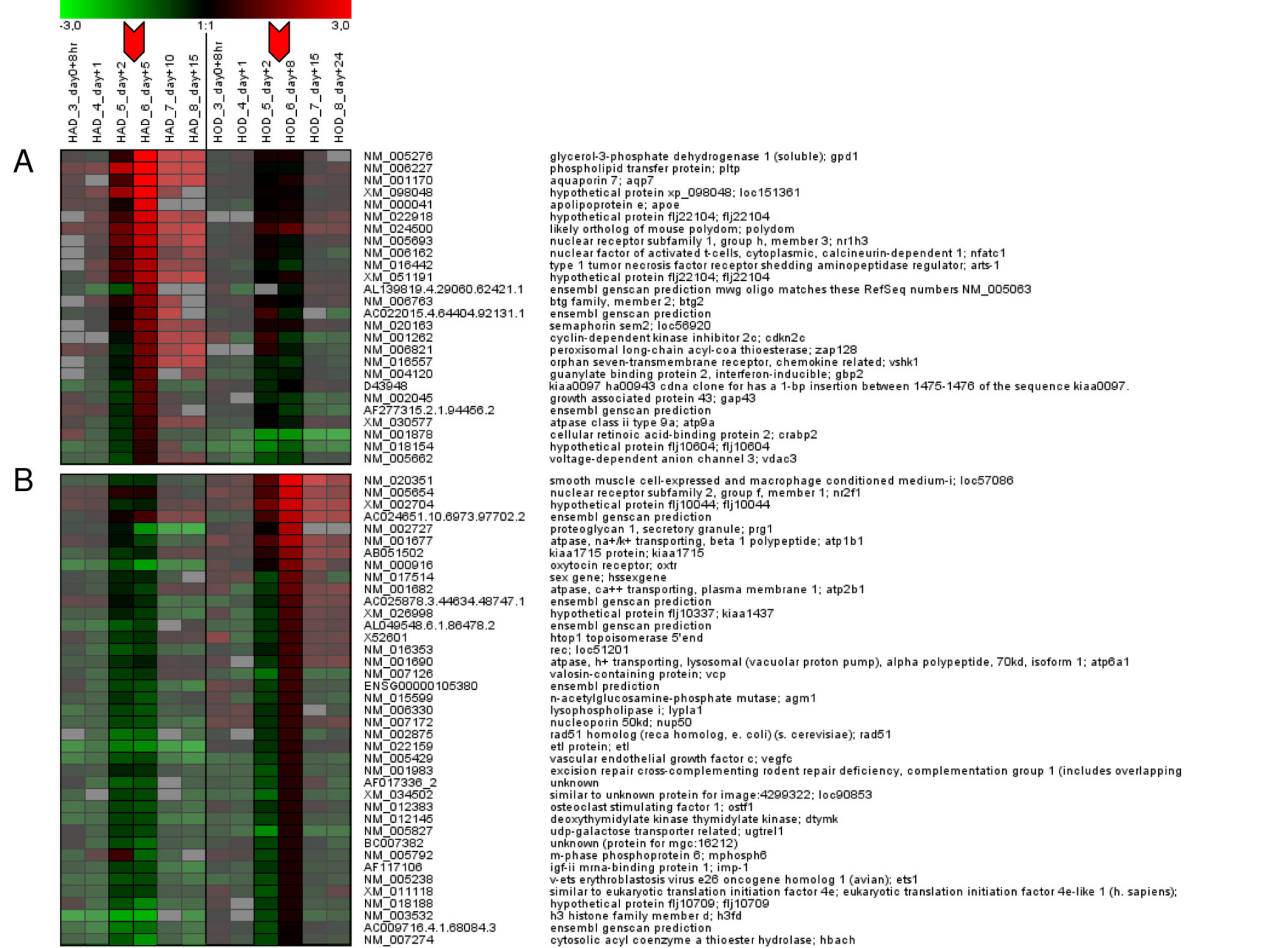
**Genes differentially expressed during commitment between time point 5 and 6.** A) 26 genes specifically up-regulated between time point 5 and 6 during adipocyte differentiation. B) 39 genes specifically up-regulated between time point 5 and 6 during osteoblast differentiation.

Genes that are differentially expressed at a specific time point may be involved in lineage commitment. We therefore extracted genes that were differentially expressed from time points 5 to 6 specifically in one differentiation pathway. In support of the time point selection, we found *TAZ*, a known transcriptional coactivator of bone development and repressor of adipocyte differentiation [34], derepressed at the time of commitment in osteoblastogenesis, but with delay in adipogenesis after the commitment. 26 genes regulated in this manner were identified for the adipogenic lineage and 39 for the osteogenic lineage (Figure 3). Interestingly, each cluster contained a nuclear receptor: *NR1H3 *(liver X receptor alpha; *LXRα*) for adipogenic commitment, and *NR2F1 *(chicken ovalbumin upstream promoter transcription factor 1; *COUP-TF1*), an orphan nuclear receptor acting predominantly as a transcription repressor [35] for osteogenic commitment. To examine co-regulation, we screened the promoter sequences available for these 65 genes for statistically over-represented transcription factor binding sites (TFBS). Significantly enriched TFBS were found in both gene clusters. For adipogenesis the promoters of 26 genes had overrepresented TFBS for 20 transcription factors and for osteoblastogenesis promoters of 39 genes had overrepresented TFBS for 11 transcription factors. (see Additional files 6 + 7). In the adipogenic commitment cluster, six genes were found to contain the *PPARγ *DR1 response element and two genes to contain an *LXR *response element, providing evidence for the potential co-regulation of these co-expressed genes. Strikingly, seven adipogenic-related genes were found to contain a *COUP-TF1 *binding site with the highest significance. In a reciprocal manner, five genes in the osteogenic commitment cluster contained a binding site for *SREBP*, a transcription factor also found to be up-regulated during early adipogenesis.

During adipogenic commitment, we identified the phospholipid transfer protein (*PLTP*), an important molecule in the regulation of phospholipid, cholesterol and HDL metabolism [36]. Interestingly, the computational analysis of the promoter of PLTP showed a *KROX *response element (see Additional file 4, significant rank #3). During the osteogenic commitment, the hypothetical protein *FLJ22104 *(*TMEM135*) was found to contain 11 significantly enriched TFBS, the highest number of TFBS in a given gene an representing more than 50% of all significant TFBS found. Based on the results of in-depth protein sequence analysis, *TMEM135 *is a multi-transmembrane protein with 7 transmembrane helices of high confidence. The N-terminal transmembrane region (3 helices) and the C-terminal region (4 helices) are separated by a mixed charged cluster (amino acid (aa): 200–250)) with a high KRED content (43%). Homologies exist with the transmembrane region of similar to frizzled 4 (XP_788346; aa: 153–447; E-value = 1E-53), a component of the Wnt signaling pathway [37]. Less extensive homologies exist to peroxisomal protein 4 (NP_757377; E-value = 6E-37; 5th psi-blast round) and *TIM17 *(pfam02466; rps-blast E-value = 0.028), a mitochondrial translocator.

### Validation studies on selected candidate genes

**Figure 4:**
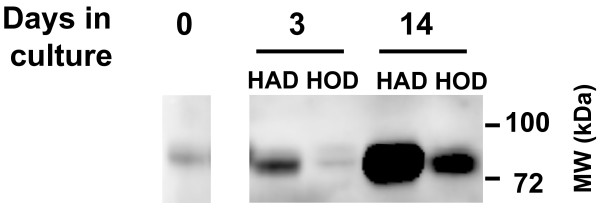
***PLTP*****secretion during differentiation of hMADS cells to adipocytes and osteoblasts.** The expression of *PLTP *in the secretion media of hMADS cells at day 0, and during differentiation at days 3 and 14 adipocytes (HAD) or osteoblasts (HOD) after 6 h of incubation.

The 4 selected candidate genes, *LXRα*, *PLTP *(adipogenesis), *COUP-TF1 *and *TMEM135 *(osteoblastogenesis), were subjected to additional validation experiments. Western blot analysis of the supernatant from differentiating hMADS after 3 days in culture revealed that *PLTP *is secreted during adipocyte differentiation but not during osteoblast differentiation (Figure 4). After two weeks, the protein expression levels were very high in the adipocyte culture medium whereas a lower level was detected in the osteoblast culture medium suggesting that adipocytes secreted *PLTP *more than osteoblasts.

**Figure 5:**
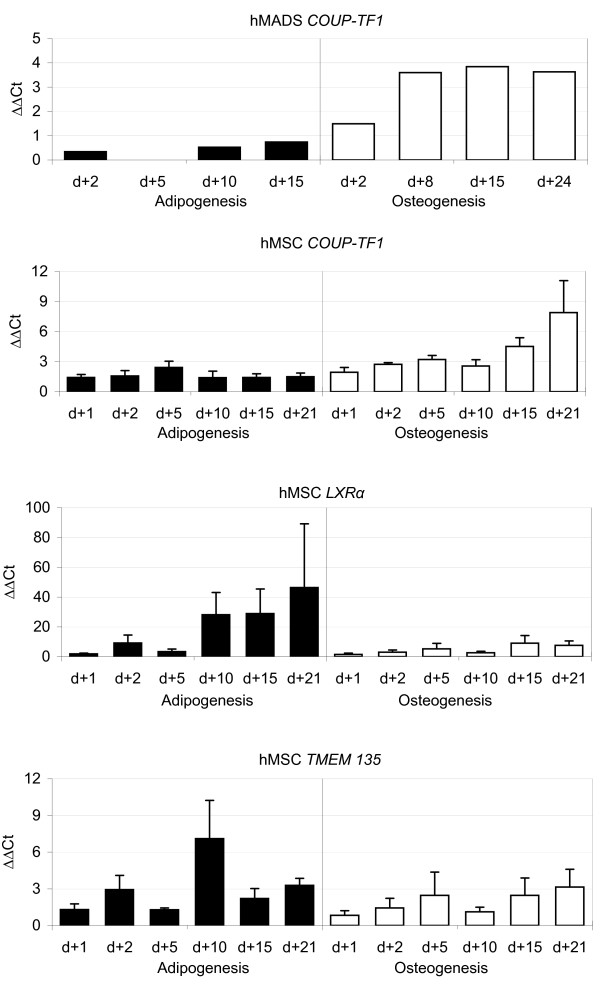
RT-PCR results for *COUP-TF1*, *LXRα*, and *TMEM135 *during adipogenesis and ostoblastogenesis from bone marrow stromal cells from healthy donors.

To obtain validation data for the other candidate genes, primary hMSC obtained from healthy donors were differentiated and expression levels analyzed by RT-PCR (Figure 5). *COUP-TF1 *exhibited higher expression levels during osteoblastogenesis. The expression level of *COUP-TF2 *was the same in both cell models and differentiation lineages (data not shown). *LXRα *was expressed at an eight-fold higher level in adipocytes than in osteoblasts and *TMEM135 *expression was on average 2-fold higher in adipocytes than in osteoblasts.

This data provide additional evidence for an involvement of the selected candidate genes, *PLTP*, *LXRα*, *COUP-TF1*, and the transmembrane protein *TMEM135 *respectively in adipocyte and osteoblast differentiation. *PLTP *was secreted at both early and late time points of adipocyte differentiation of hMADS. *LXRα*, *COUP-TF1*, and *TMEM135 *were transcriptionally activated in differentiating bone marrow stromal cells from healthy donors in a lineage-dependent manner.

## Discussion

In this study we have used a unique model and large-scale gene expression profiling to study the transcriptional regulation of biological processes occurring during the differentiation of multipotent stem cells. The data presented here and the bioinformatics analyses considerably augment previous studies of early adipocyte and osteoblast differentiation. Our comprehensive and unbiased approach resulted in the identification of 1606 differentially expressed genes and suggests several important biological conclusions.

Firstly, the gene down-regulation was most prevalent in both adipogenesis and osteoblastogenesis. The extent of gene repression varied before and after commitment and was more pronounced in osteoblastogenesis. Surprisingly, GO term analysis showed that only few significantly enriched biological processes were transcriptionally up-regulated prior to commitment. This contrasts with our previous study in which many molecular processes were found to be modulated [24]. The results of the current study suggest that repression of diverse sets of genes is a general phenomenon during early differentiation of stem cells. It is also tempting to speculate that committed cells can undergo transdifferentiation because of the lack of activation of specific cellular processes. It has been shown for example that white adipocytes can be transdifferentiated to brown adipocytes [3]. Further studies are necessary to identify both the length of the time window for the transdifferentiation and the appropriate stimuli.

Secondly, computational analyses of the 3'UTR of the differentially expressed genes support the hypothesis that gene repression is modulated by miRNAs. Several miRNAs have been shown to affect late stages of adipocyte differentiation, and the expression of other miRNAs has been reported during osteoblast differentiation [38-41]. Here we provide data showing that miRNAs might be involved in gene regulation during the early stages of both lineages. Using conservative criteria for the miRNA binding sites, over 80% of the genes with unique 3'UTR were found to contain at least one exact antisense match for the 7-nt seed and are thus potential target for 30 miRNAs. Although the number of false positives in this analysis is difficult to estimate, it is likely that many miRNAs repress a large number of genes. This observation has implications for therapeutic targeting using antisense strategies. Further studies will be required to identify the most promising candidates.

Thirdly, gene expression data and promoter analysis of the differentially expressed genes identified 26 that may play a role in adipogenic and 39 in osteoblastogenic commitment. From these, we selected four candidates: *LXRα*, and *PLTP *for adipogenesis, and *COUP-TF1 *and *TMEM135 *for osteoblastogenesis. Validation studies provided additional evidence that these genes are involved in determining the balance between adipocyte and osteoblast differentiation.

The nuclear hormone receptor and transcription factor, *LXRα*, is known to play a major role in the regulation of cholesterol catabolism, inflammatory gene expression and the switch between glucose metabolism and fatty acid synthesis [42-44]. *PLTP *is believed to be a direct target gene of *LXR*s which is consistent with our results demonstrating specific activation of *LXRα *and *PLTP *at the time of commitment and secretion of *PLTP *in a differentiation-dependent manner [45]. The finding that *PLTP *contains a *KROX *response element as a significantly enriched TFBS and the very early transcriptional up-regulation of *PLTP *compared to *LXRα *however indicates that *PLTP *is also activated by additional, *LXRα*-independent regulatory mechanisms.

Our finding that *COUP-TF1 *is transcriptionally activated at the time of osteogenic commitment is consistent with loss of function studies in mice revealing bone fusion in occipital bones in 98% of the null mutants [46]. Transcriptional repression by the orphan receptor *COUP-TF1 *is known to play a critical role in several developmental processes including neurogenesis, angiogenesis and heart development [35]. Strikingly, we identified its binding site in seven genes of the adipogenic commitment cluster with the highest significance suggesting a major role of *COUP-TF1 *in lineage commitment involving suppression of adipogenic and promotion of osteogenic differentiation. Interestingly, in a recent study it was shown that *COUP-TF2 *suppresses adipogenesis [47]. It should be noted that in this study committed mouse cells were used whereas in our study uncommitted human cells were investigated. Given this experimental evidence in differing species and commitments, and the high degree of similarity to binding sites for the *COUP-TF*s, we hypothesize that the orphan nuclear receptor family *COUP-TF *are critical regulators of adipogenesis and osteoblastogenesis. The identification of exogenous *COUP-TF *ligands will enable the exploration of therapeutic opportunities involving modulation of *COUP-TF*.

Finally, we have also investigated the role of *TMEM135*, a gene previously identified in a human lung adenocarcinoma cell line cDNA library [48] in osteogenesis. Based on its sequence structure and homologies with other gene products, *TMEM135 *is likely to be a channel (charged cluster in the middle) or a receptor (charged cluster to the surface). With the highest number of significantly enriched TFBS, e.g. for *COUP-TF1*, *PPARγ*, *KROX*, *GATA-3*, and *LXR*, *TMEM135 *is an interesting candidate for involvement in lineage commitment, an hypothesis that must nevertheless be validated by further studies.

In conclusion, our study on gene expression signatures during adipogenic and osteoblastogenic differentiation of hMADS cells showed common global transcriptional patterns for both lineages. Our results reveal gene repression as a predominant early mechanism before final cell commitment indicating that gene repression may have a larger functional role in controlling the cell fate. This repression is likely to be controlled by miRNAs. We were moreover able to identify 65 genes as candidates for genes controlling the adipocyte/osteoblast balance and to further evaluate four of these. Additional studies will explore the precise role of these candidate genes in regulating the adipogenesis/osteoblastogenesis switch.

## Methods

### hMADS cell culture

hMADS cells were grown in Dulbecco's Modified Eagle's Medium (DMEM low glucose) containing 10% fetal calf serum (FCS), and 100 U/ml penicillin and streptomycin. After reaching 80% confluence, adherent cells were detached with 0.25% trypsin EDTA and seeded at a density of 4500 cells per cm^2^. hMADS cells were maintained in proliferation medium supplemented with 2 ng/ml fibroblast growth factor 2 (*FGF2*) [21].

For adipocyte differentiation, two days after confluence (referred to as day 0), cells were cultured in DMEM/Ham's F12 medium supplemented with 10 μg/ml transferrin, 0.86 μM insulin, 0.2 nM of triiodothyronine, 1 μM dexamethasone, 100 μM isobutyl-methylxanthine, and 100 nM rosiglitazone. Three days later, the medium was changed (dexamethasone and isobutyl-methylxanthine were omitted). Neutral lipid accumulation was assessed by oil red O staining as described previously [49].

For osteoblast differentiation, cells were cultured for 24 days in α-MEM containing 10% FCS, 50 μg/ml L-ascorbic acid phosphate, 10 mM β-glycerophosphate, and 100 nM dexamethasone. Alizarin red staining was performed as previously described [50]. All media were changed every other day.

### hMSC cell culture

The mononuclear cell fractions were derived from bone marrow from four donors who gave consent after full information and approval by the hospital ethical committee (No. 12-091). hMSC were grown in Dulbecco's modified Eagle's medium (high glucose) containing 10% fetal bovine serum PAN, 2 mM L-glutamine, 100 U/ml penicillin and streptomycin, 100 μg/ml normocin (InvivoGen) under standard conditions. Adipocyte differentiation was induced on the third day after the cells reached confluence with medium containing 1 μM dexamethasone, 0.5 mM isobutylmethylxantine, 60 μM indomethacine, 1 μM rosiglitazone and 10 μg/ml insulin. After 2–3 days the cells were refed with medium was containing only 10 μg/ml insulin for a period of 24 h. This cycle was repeated a total of 3 times. During the final 7 days of differentiation the cells were cultured in medium containing 10 μg/ml insulin. Differentiation was confirmed by oil red O staining.

Osteoblast differentiation was triggered 24 h after cell seeding with medium containing 0.1 μM dexamethasone, 10 mM glycerophosphat and 100 mM L-ascorbic acid phosphate. The different time period compared to hMADS is due to the differences of the origins of cells (adipose tissue vs. bone marrow) and the differences in the the cultivation media. The medium was changed 2–3 times a week over a total period of 21 days. Differentiation was confirmed by alizarin red staining.

### Sample preparation and microarray hybridization

Three independent cell culture experiments were performed as biological replicates for both adipocyte and osteoblast differentiation. Cells were harvested at the pre-confluent stage as reference and at eight subsequent time points (adipocyte differentiation: day -2 (tp1) and 0 (tp2) before differentiation induction, and 8 hours (tp3), 1 (tp4), 2 (tp5), 5 (tp6), 10 (tp7), and 15 days (tp8) after induction of differentiation; osteoblast differentiation: day -2 (tp1) and 0 (tp2) before differentiation induction, and 8 hours (tp3), 1 (tp4), 2 (tp5), 8 (tp6), 15 (tp7), and 24 days (tp8) after induction of differentiation). For each time point of each independent experiment RNA was pooled from 6 different 100 mm culture dishes. Reference RNA was harvested from 48 dishes of cells at the preconfluent stage.

Total RNA was isolated using TRIzol reagent (Invitrogen, Carlsbad, CA, USA) or Tri-Reagent (Euromedex, Mundolsheim, France) according to the manufacturer's instructions. The quality of the RNA was checked using Agilent 2100 Bioanalyzer RNA assays (Agilent Technologies, Palo Alto, CA, USA). 20 μg of total RNA was used for indirect labeling with Cy3 and Cy5. Human microarrays spotted with 29550 oligonucleotides and the represented genes identified with RefSeq IDs were produced and hybridized as previously described [51]. All hybridizations were repeated with reversed dye assignment (dye-swap). Hybridized slides were scanned with a GenePix 4000B microarray scanner (Axon Instruments, Sunnyvale, CA, USA) at 10 μm resolution and the resultant TIFF images analysed with GenePix Pro 4.1 software (Axon Instruments).

### Real-time RT-PCR

41 genes indicative for the adipogenic lineage, 22 genes indicative for the osteogenic lineage, and 37 genes potentially involved in self-renewal have been validated by reverse transcription polymerase chain reaction (RT-PCR) analysis of total RNA as described previously [20]. An aliquot of the PCR product was analyzed on a 2% ethidium bromide-stained agarose gel. For quantitative PCR, the final reaction volume was 25 μl, including specific primers (0.2–0.4 μM), 5–12 ng of reverse-transcription product and 9–12.5 μl of SYBR green master mix (Eurogentec, Angers, France). Quantitative PCR was carried out as follows: 2 minutes at 50°C; 10 minutes at 95°C; and 35 cycles of 15 seconds at 95°C and 1 minute at 60°C. Real-time PCR assays were run on an ABI Prism 7000 real-time PCR machine (Applied Biosystems, Foster City, CA, USA). Efficiency was estimated using LinReg software (reference). Relative expression was calculated with the ddCt method [52].

### Western blotting

After 6 hours of incubation for each condition, secretion media (6 ml, corresponding to 2 dishes of 100 mm diameter) were collected on ice, centrifuged, filtered to remove cell debris and supplemented with complete protease inhibitor cocktail. The samples were concentrated before analysis by ultra-filtration (Millipore, Centricon, 5 kDa cut-off). Equal volumes of secretion media were separated by SDS-PAGE, transferred to PVDF membrane and processed for the expression of *PLTP *using specific anti-human antibodies (provided by M. Jauhiainen) [53]. Immunoreactive signal was visualized with the ECL chemiluminescence detection kit (Amersham) according to the manufacturer's instructions.

### RNA isolation

Cells were collected in TRIzol (Invitrogen) before and 24 h, 48 h, 5, 10, 15, 21 days after differentiation induction. Total RNA was isolated according to the manufacturer's instructions. The quality of the RNA was examined using Agilent 2100 Bioanalyzer RNA assays (Agilent Technologies).

### Microarray data analysis

Global mean and dye swap normalization were applied using ArrayNorm [54]. The resulting ratios were log2 transformed and averaged over three independent experiments. All experimental parameters, images, and raw and transformed data were uploaded to the microarray database MARS [55] and submitted via MAGE-ML export to a public repository (ArrayExpress [56], accession number A-MARS 3 and E-MARS 10).

### Statistical analysis

The aim was to identify genes with a significant change in expression before induction (tp1 and tp2) compared to the expression level after induction (time points 3 to 8). To that end we calculated the difference in expression between time point 1 and all time points after induction (tp3.tp8), and the same was done for time point 2. For each gene, we considered the maximum of all these differences (in absolute values) as an estimate of its change in expression before and after induction. If that log-ratio was larger than +/-1 (2 fold change) we consider the gene as differentially expressed after differentiation induction. We categorized these differentially expressed genes according to the following profiles: a) Opposite expression, b) lineage-specific expression and c) similar expression in the adipocyte and osteoblast differentiation pathways. For those genes differentially expressed in either HAD or HOD experiments, the Pearson correlation coefficient (scale-invariant) was calculated and its significance was assessed using the permutation test implemented in the R permax package http://www.r-project.org. This analysis yielded a total of eight clusters: three for adipogenic-related genes (ARG) (HAD-HOD; 1: up-down; 2: up-non; 3: down-non), three clusters for osteogenic-related genes (ORG) (HAD-HOD; 4: down-up; 5: non-up; 6: non-down), and two clusters for differentiation-independent genes (DIG) (HAD-HOD; 7: up-up; 8: down-down).

### Gene Ontology classification

Gene Ontology (GO) terms for biological processes were derived from the Gene Ontology database (Gene Ontology Consortium) using the RefSeq accession numbers. All cluster analyses and visualizations were performed using Genesis [57].

### Identification of miRNA target sites in the 3'-UTR

All available 3'-UTR sequences (23,611) for human genes were extracted using the human genome version hg18 (NCBI build 36.1) from the USCS Genome Browser [58] and genomic coordinates for human genes from the Refseq database [59]. A total of 470 human miRNA sequences were derived from mirBase [60]. The 3'-UTR sequences were searched for perfect antisense matches to the designated seed region of each miRNA (bases 2–8 from the 5' end). Significantly over-represented miRNA motifs in the 3'-UTRs of genes in each cluster and for the full set of differentially regulated genes that were significantly over-represented compared with the motifs in the whole 3'-UTR sequence set were determined using the one-sided Fisher's exact test (*p *< 0.05). To account for multiple testing p-values were adjusted controlling for the false discovery rate (FDR < 5%) as proposed by Benjamini and Hochberg [61].

### Promoter analysis

Promoter analysis was performed using CRSD, a comprehensive web server for composite regulatory signature discovery [62]. Iterative enrichment analysis was conducted based on putative promoter and TRANSFAC databases. Transcription factor binding sites were selected as statistically over-represented with a significance level of the corrected P-value < 0.1.

### Protein domain analysis

Protein sequences were annotated de novo using the more than 40 prediction tools that are integrated in the ANNOTATOR, a novel protein sequence analysis system including composition, TM region, motif and homology analysis [63,64].

## Authors' contributions

MS designed and performed the experiments, and analyzed and interpreted the data. CE and L–EZ participated to most biological and validation experiments and analyzed the data. CC collected hMADS secretion media and performed Western blot analysis for PLTP expression. SY hybridized the microarrays. KD and AP performed the qPCR analysis of the primary cells. HH performed the bioinformatics analyses. FS–C was responsible for the statistical analyses. GF isolated the bone marrow aspirates. CP, AP, and MS developed and produced the microarrays. RW was responsible for the clinical studies. GA participated in analysing and interpretating the data. CD participated to design of cell experiments and analysis of the data. E–ZA participated to conception of the research program and data analysis. ZT was responsible for the overall conception, project coordination, and data interpretation.

## Supplementary Material

Additional file 1 Real-time RT-PCR of 19 adipogenic and 14 osteogenic marker genes during adipocyte and osteoblast differentiation. A) Expression profiles of 21 adipogenic marker genes during adipocyte differentiation are displayed for the time points 5 (day+2), 7 (day+10), and 8 (day+15) after differentiation induction; B) Expression profiles of 14 osteogenic marker genes during osteoblast differentiation are displayed for the time points 5 (day+2), 7 (day+15), and 8 (day+24) after differentiation induction. Click here for file

Additional file 2 Real-time RT-PCR and microarray data. Real-time RT-PCR and microarray data of 41 adipogenic, 22 osteogenic marker genes, and 37 potential self renewal-related genes with common profile during adipocyte and osteoblast differentiation. Click here for file

Additional file 3 Heat map of expression data. Expression values for 1,606 genes in eight distinct clusters. Click here for file

Additional file 4 miRNA motifs in up- and down-regulated gene clusters. Number of miRNA motifs in up- and down-regulated gene clusters in both lineages before and after commitment. Click here for file

Additional file 5 Significantly over-represented miRNA motifs. Significantly over-represented miRNA motifs. Click here for file

Additional file 6 Significantly over-represented transcription factor binding sites. Significantly over-represented transcription factor binding sites of 26 genes with a specific profile for the adipogenic commitment. Click here for file

Additional file 7 Significantly over-represented transcription factor binding sites. Significantly over-represented transcription factor binding sites of 39 genes with a specific profile for the osteogenic commitment. Click here for file
