# Supplementary figures and images for: Comparative transcriptomics of human multipotent stem cells during adipogenesis and osteoblastogenesis
Source: BMC Genomics. 2008 Jul 17;9:340. doi: 10.1186/1471-2164-9-340 (PMC2492879; doi:10.1186/1471-2164-9-340)

Additional file 1

A

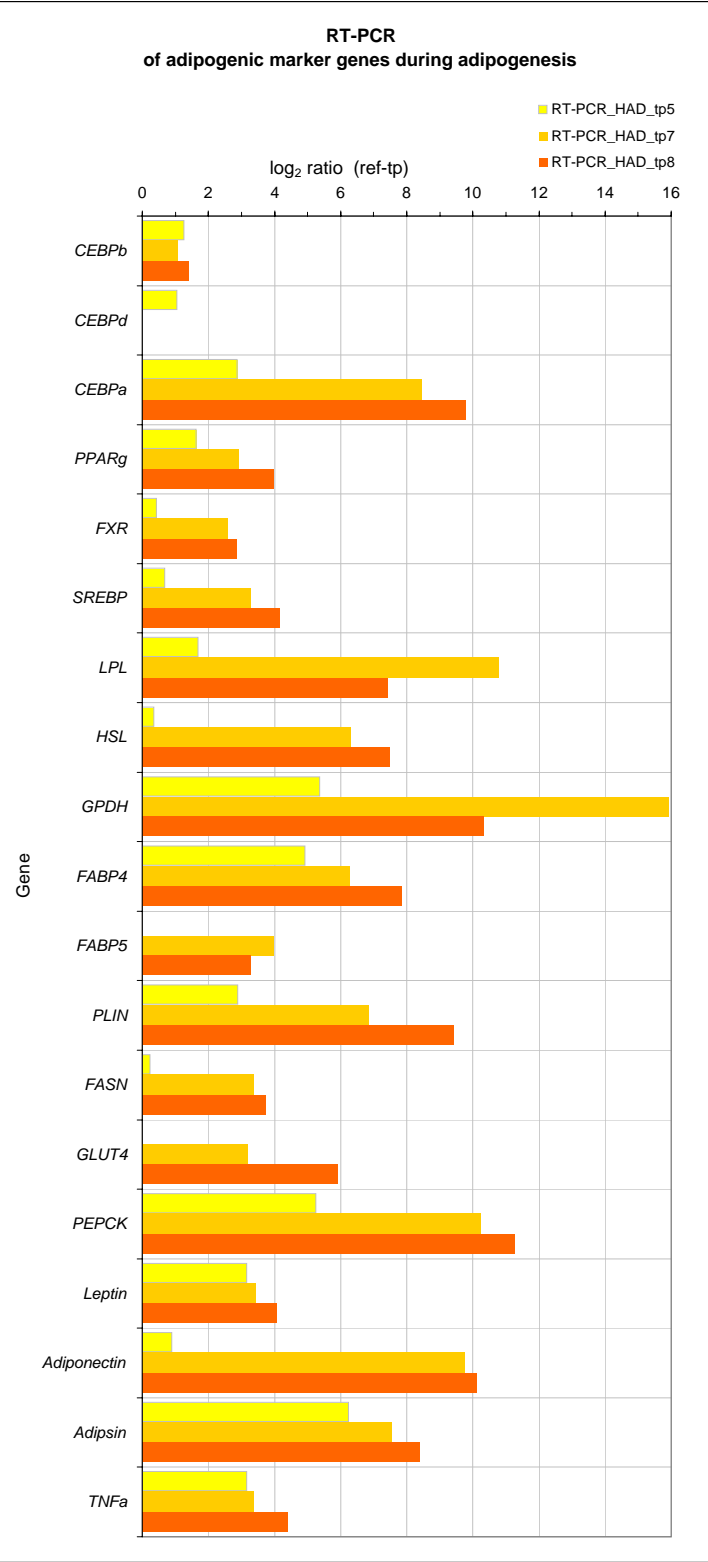

B

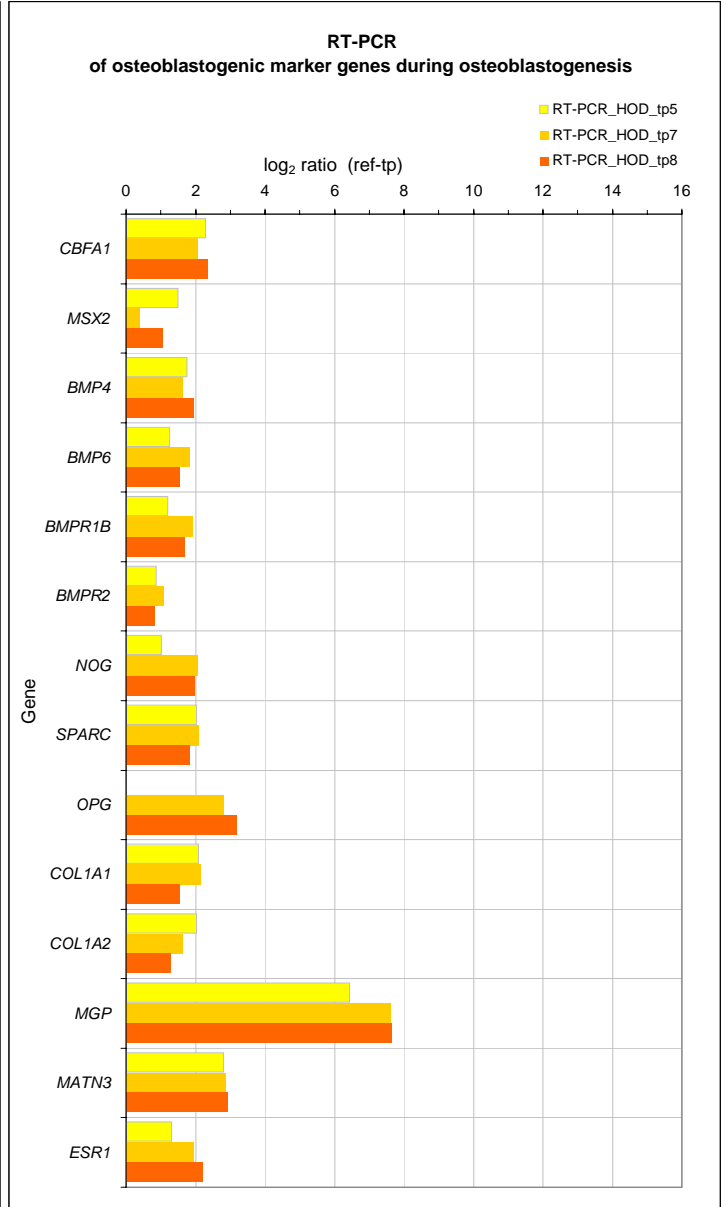

Supplement: Additional file 1 — Real-time RT-PCR of 19 adipogenic and 14 osteogenic marker genes during adipocyte and osteoblast differentiation. A) Expression profiles of 21 adipogenic marker genes during adipocyte differentiation are displayed for the time points 5 (day+2), 7 (day+10), and 8 (day+15) after differentiation induction; B) Expression profiles of 14 osteogenic marker genes during osteoblast differentiation are displayed for the time points 5 (day+2), 7 (day+15), and 8 (day+24) after differentiation induction. [file 1471-2164-9-340-S1.pdf]

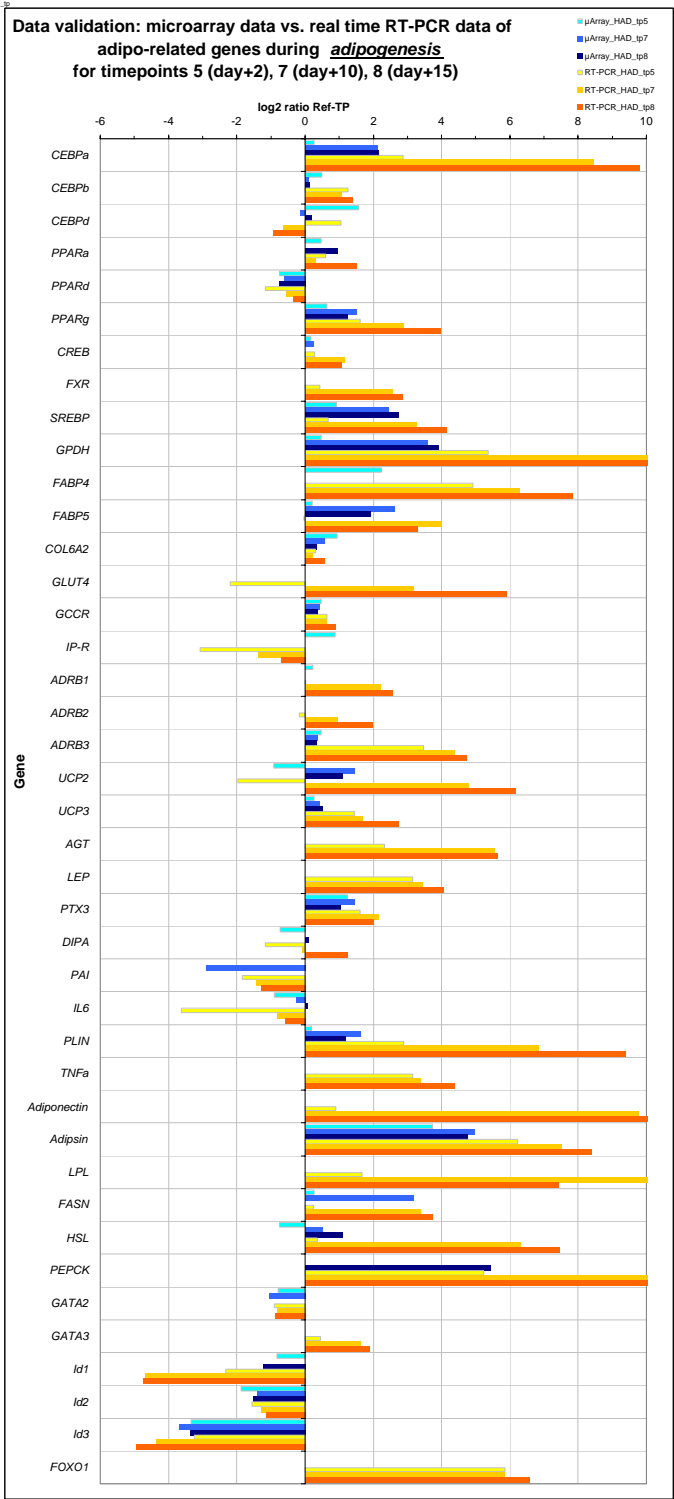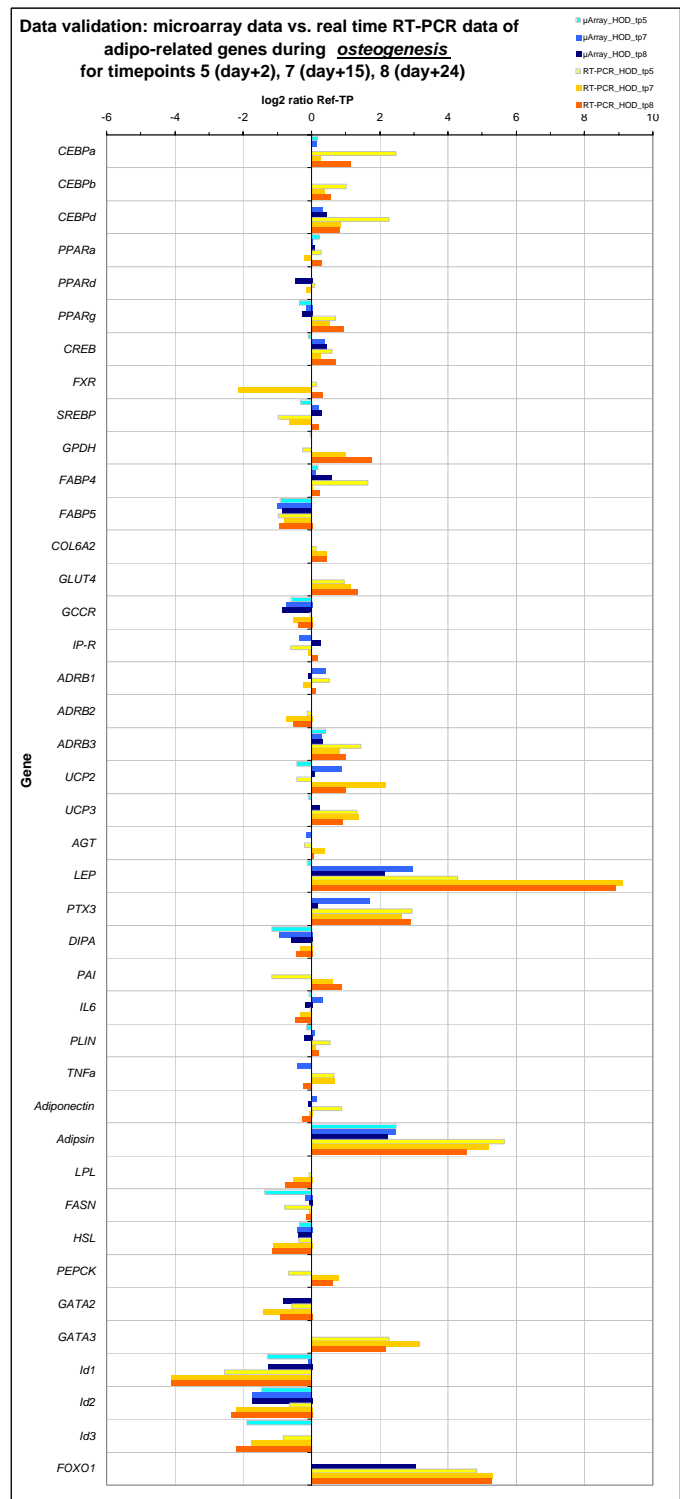

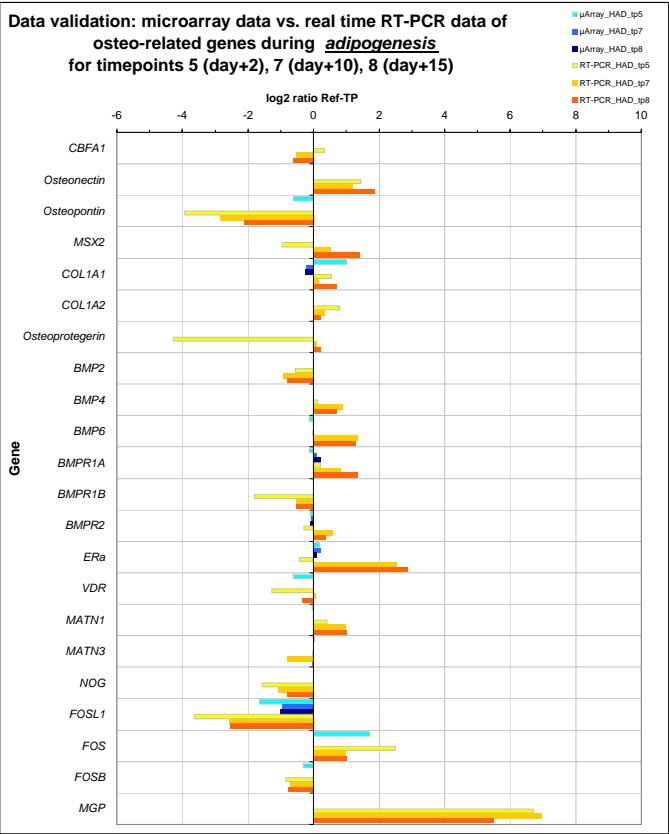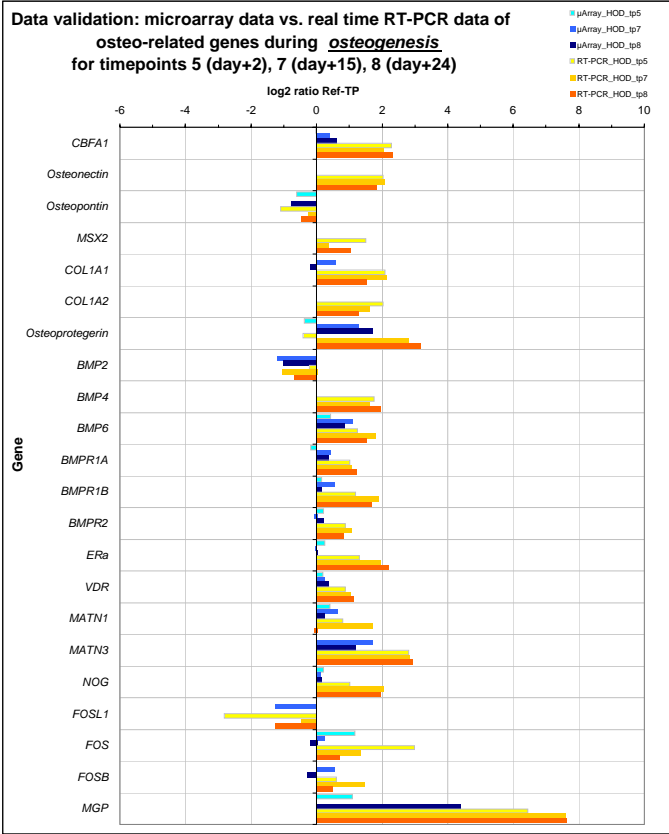

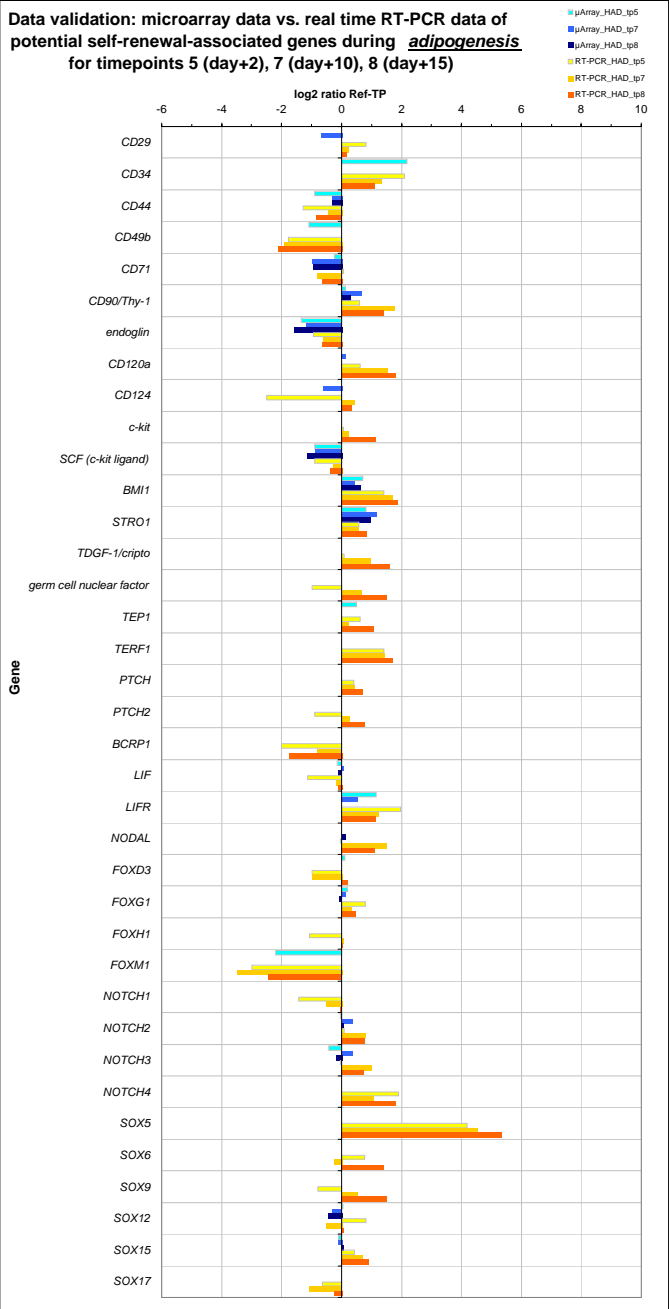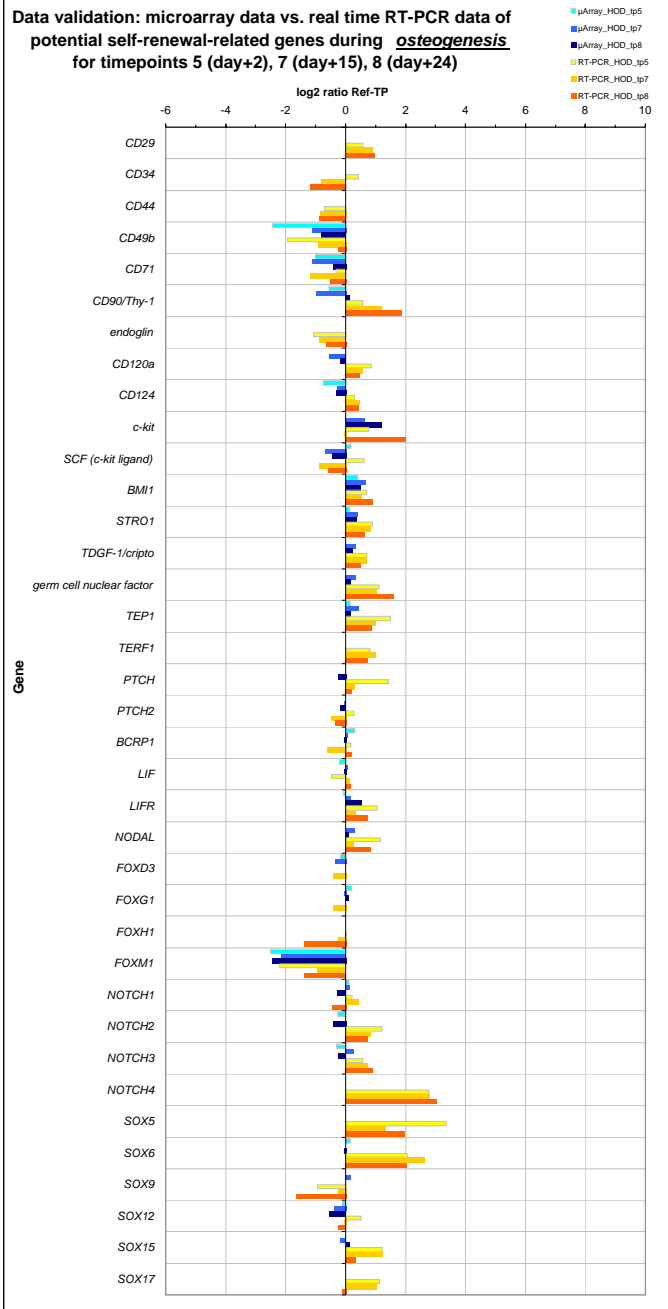

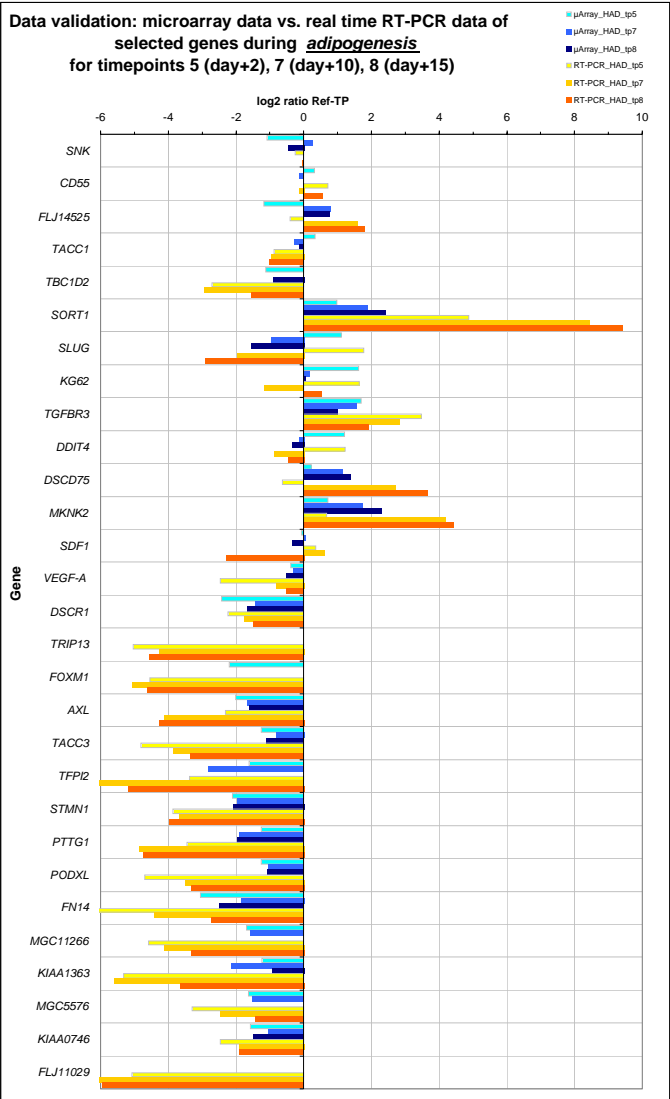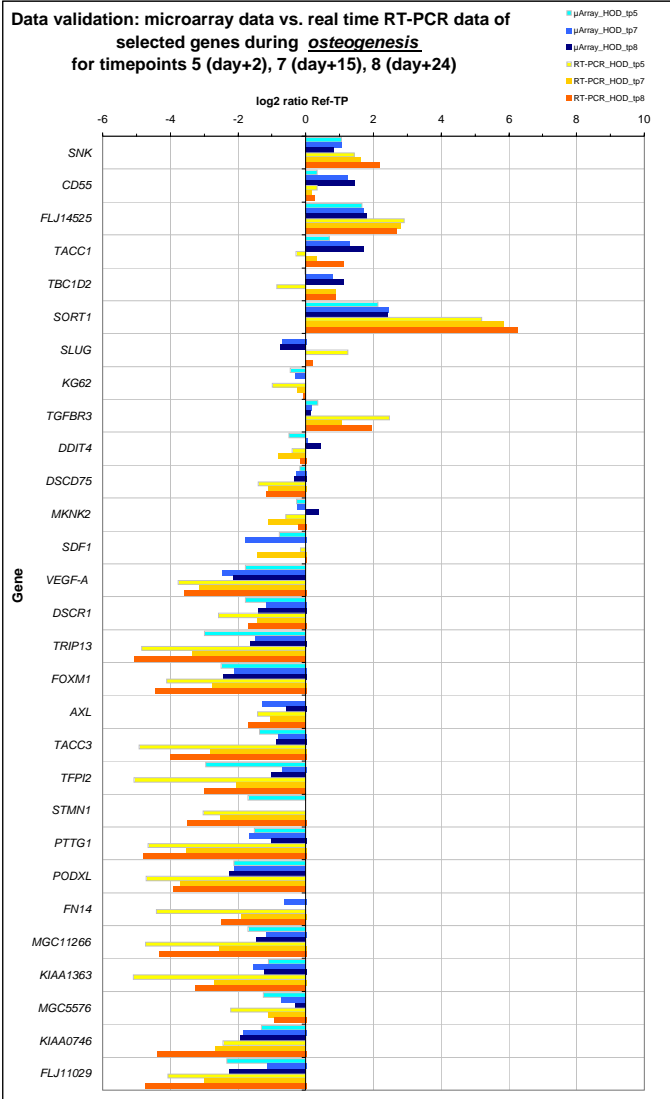

Supplement: Additional file 2 — Real-time RT-PCR and microarray data. Real-time RT-PCR and microarray data of 41 adipogenic, 22 osteogenic marker genes, and 37 potential self renewal-related genes with common profile during adipocyte and osteoblast differentiation. [file 1471-2164-9-340-S2.pdf]

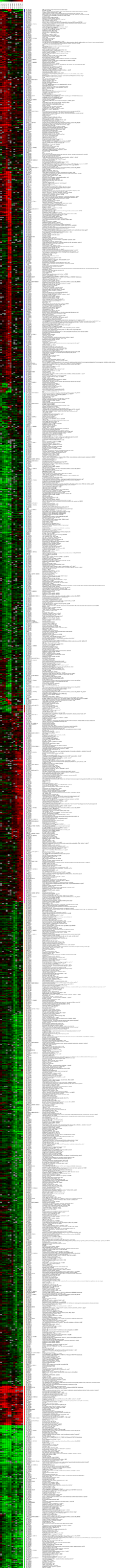

Supplement: Additional file 3 — Heat map of expression data. Expression values for 1,606 genes in eight distinct clusters. [file 1471-2164-9-340-S3.png]

## Additional file 4

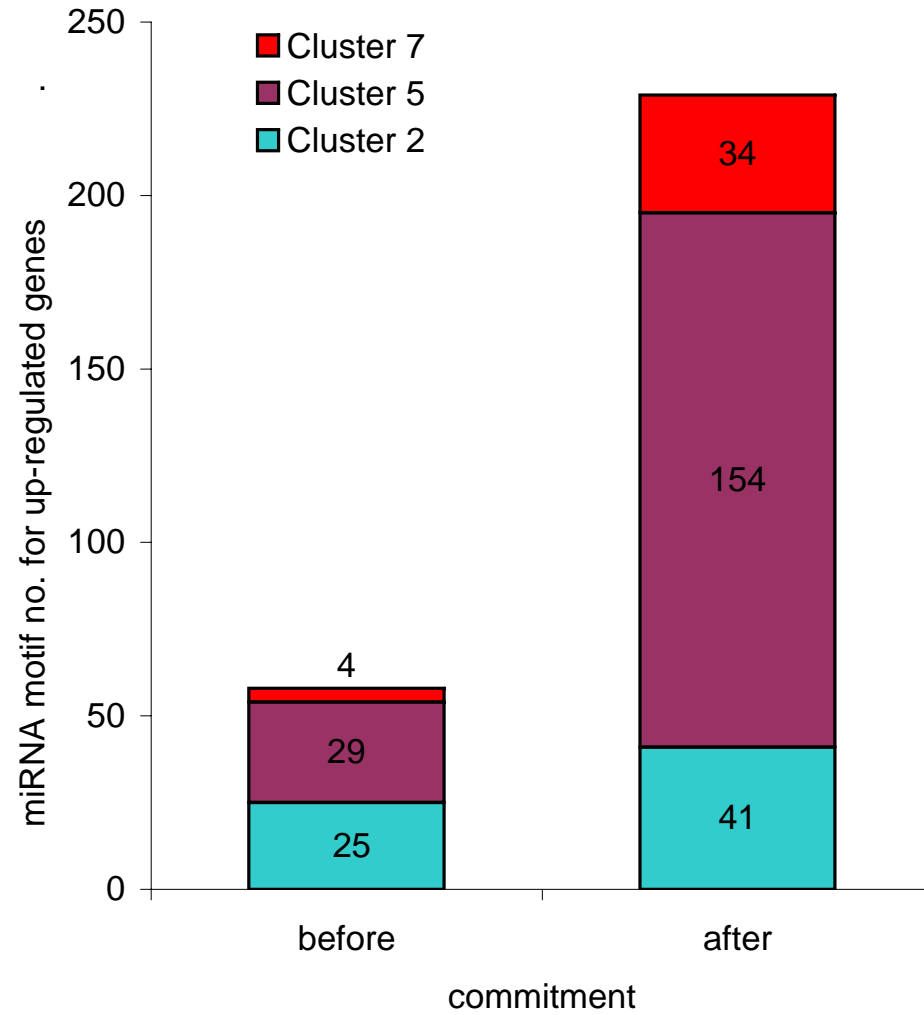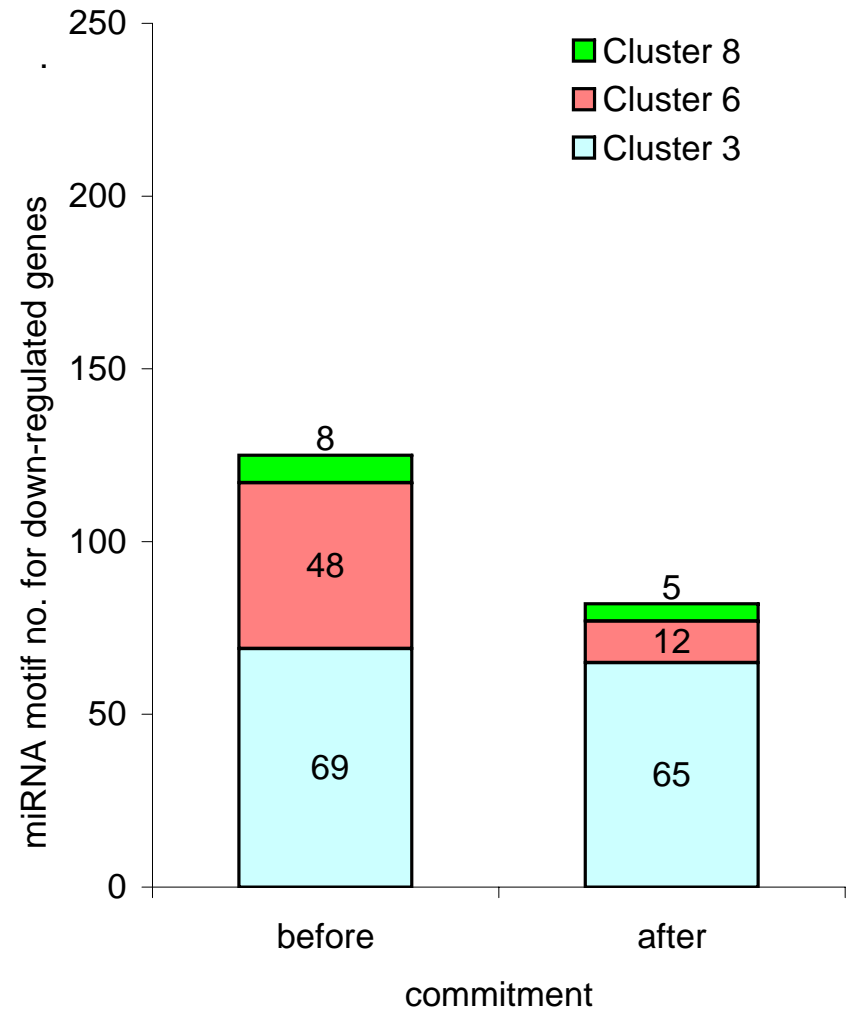

Supplement: Additional file 5 — Significantly over-represented miRNA motifs. Significantly over-represented miRNA motifs. [file 1471-2164-9-340-S5.pdf]
