# Supplementary material for: Comparative transcriptomics of human multipotent stem cells during adipogenesis and osteoblastogenesis
Source: BMC Genomics. 2008 Jul 17;9:340. doi: 10.1186/1471-2164-9-340 (PMC2492879; doi:10.1186/1471-2164-9-340)
Supplement: Additional file 4 — miRNA motifs in up- and down-regulated gene clusters. Number of miRNA motifs in up- and down-regulated gene clusters in both lineages before and after commitment. [file 1471-2164-9-340-S4.pdf]

Additional file 5

Significantly over-represented miRNA motifs

Rank	microRNA	# genes with microRNA seed match (2-8) of entire sequence set	# genes with microRNA seed match (2-8) of differentially expressed gened with 3'UTR	p-value	adjusted p-value (FDR)
1	hsa-miR-30a-3p	3120	209	5.30E-06	0.0012
2	hsa-miR-30e-3p	3120	209	5.30E-06	0.0012
3	hsa-miR-548c	5001	309	1.32E-05	0.0021
4	hsa-miR-194	1667	120	4.56E-05	0.0046
5	hsa-miR-506	2469	166	4.92E-05	0.0046
6	hsa-miR-605	2061	141	9.31E-05	0.0047
7	hsa-miR-124a	2221	149	0.0001	0.0047
8	hsa-miR-202*	1960	135	0.0001	0.0047
9	hsa-miR-320	3065	196	0.0001	0.0047
10	hsa-miR-381	2025	138	0.0001	0.0047
11	hsa-miR-548d	3856	237	0.0003	0.0128
12	hsa-miR-579	4938	289	0.0010	0.0382
13	hsa-miR-641	2672	167	0.0012	0.0382
14	hsa-miR-520g	3619	218	0.0013	0.0382
15	hsa-miR-520h	3619	218	0.0013	0.0382
16	hsa-miR-570	5111	297	0.0013	0.0382
17	hsa-miR-495	3515	212	0.0015	0.0396
18	hsa-miR-19a	2377	150	0.0016	0.0396
19	hsa-miR-19b	2377	150	0.0016	0.0396
20	hsa-miR-216	2352	148	0.0019	0.0400
21	hsa-miR-653	3237	196	0.0019	0.0400
22	hsa-miR-106a	3551	212	0.0023	0.0400
23	hsa-miR-106b	3551	212	0.0023	0.0400
24	hsa-miR-17-5p	3551	212	0.0023	0.0400
25	hsa-miR-20a	3551	212	0.0023	0.0400
26	hsa-miR-20b	3551	212	0.0023	0.0400
27	hsa-miR-519d	3551	212	0.0023	0.0400
28	hsa-miR-199a*	1780	115	0.0028	0.0470
29	hsa-miR-425-5p	1498	99	0.0029	0.0470
30	hsa-miR-607	3913	230	0.0031	0.0486
