# Supplementary material for: Comparative transcriptomics of human multipotent stem cells during adipogenesis and osteoblastogenesis
Source: BMC Genomics. 2008 Jul 17;9:340. doi: 10.1186/1471-2164-9-340 (PMC2492879; doi:10.1186/1471-2164-9-340)
Supplement: Additional file 6 — Significantly over-represented transcription factor binding sites. Significantly over-represented transcription factor binding sites of 26 genes with a specific profile for the adipogenic commitment. [file 1471-2164-9-340-S6.pdf]

Additional file 6

Significantly over-represented transcription factor binding sites (TFBS) of 26 genes with a specific profile for the adipogenic commitment.

Rank	Transcription Factor Name (Matrix ID)	Total	Found	P-Value	Q-Value	UniGeneID	Gene Symbol	Gene Name
1	COUPTF (V$COUPTF_Q6)	1872	7	1.95474E-06	0.000662657	Hs.134974	GAP43	Growth associated protein 43
						Hs.515465	APOE	Apolipoprotein E
						Hs.188591	FLJ22104	Hypothetical protein FLJ22104
						Hs.446685	ZAP128	Acyl-CoA thioesterase 2
						Hs.438863	NR1H3	Nuclear receptor subfamily 1, group H, member 3
						Hs.201253	ch-TOG	Cytoskeleton associated protein 5
						Hs.405662	CRABP2	Cellular retinoic acid binding protein 2
2	PPAR, HNF-4, COUP, RAR (V$DR1_Q3) Direct repeat 1	1835	6	2.68424E-05	0.004549783	Hs.134974	GAP43	Growth associated protein 43
						Hs.455323	AQP7	Aquaporin 7
						Hs.188591	FLJ22104	Hypothetical protein FLJ22104
						Hs.59729	LOC56920	Sema domain, immunoglobulin domain (Ig), short basic domain, secreted, (semaphorin) 3G
						Hs.438863	NR1H3	Nuclear receptor subfamily 1, group H, member 3
						Hs.405662	CRABP2	Cellular retinoic acid binding protein 2
3	KROX (V$KROX_Q6)	1597	5	0.000176864	0.019985633	Hs.519162	BTG2	BTG family, member 2
						Hs.534074	NFATC1	Nuclear factor of activated T-cells, cytoplasmic, calcineurin-dependent 1
						Hs.439312	PLTP	Phospholipid transfer protein
						Hs.515465	APOE	Apolipoprotein E
						Hs.188591	FLJ22104	Hypothetical protein FLJ22104
4	TTF-1 (V$TTF1_Q6)	1911	5	0.000405312	0.027480171	Hs.491597	VDAC3	Voltage-dependent anion channel 3
						Hs.439312	PLTP	Phospholipid transfer protein
						Hs.26516	ASF1B	ASF1 anti-silencing function 1 homolog B (S. cerevisiae)
						Hs.188591	FLJ22104	Hypothetical protein FLJ22104
						Hs.436186	ARTS-1	Type 1 tumor necrosis factor receptor shedding aminopeptidase regulator
5	GATA-3 (V$GATA3_03) GATA-binding factor 3	1871	5	0.000367819	0.031172661	Hs.188591	FLJ22104	Hypothetical protein FLJ22104
						Hs.59729	LOC56920	Sema domain, immunoglobulin domain (Ig), short basic domain, secreted, (semaphorin) 3G
						Hs.438863	NR1H3	Nuclear receptor subfamily 1, group H, member 3
						Hs.386567	GBP2	Guanylate binding protein 2, interferon-inducible
						Hs.405662	CRABP2	Cellular retinoic acid binding protein 2
6	c-Rel (V$CREL_01) c-Rel	1913	4	0.003833323	0.056499841	Hs.534074	NFATC1	Nuclear factor of activated T-cells, cytoplasmic, calcineurin-dependent 1
						Hs.188591	FLJ22104	Hypothetical protein FLJ22104
						Hs.386567	GBP2	Guanylate binding protein 2, interferon-inducible
						Hs.436186	ARTS-1	Type 1 tumor necrosis factor receptor shedding aminopeptidase regulator
7	Barbie Box (V$BARBIE_01) barbiturate-inducible element	1898	4	0.003726897	0.0574281	Hs.134974	GAP43	Growth associated protein 43
						Hs.455323	AQP7	Aquaporin 7
						Hs.201253	ch-TOG	Cytoskeleton associated protein 5
						Hs.405662	CRABP2	Cellular retinoic acid binding protein 2
8	COUP-TF, HNF-4 (V$COUP_01)	1896	4	0.003712863	0.05993621	Hs.455323	AQP7	Aquaporin 7
						Hs.446685	ZAP128	Acyl-CoA thioesterase 2
						Hs.386567	GBP2	Guanylate binding protein 2, interferon-inducible
						Hs.405662	CRABP2	Cellular retinoic acid binding protein 2
9	HNF-4 (V$HNF4_01) hepatic nuclear factor 4	1892	4	0.003684902	0.062459089	Hs.519162	BTG2	BTG family, member 2
						Hs.455323	AQP7	Aquaporin 7
						Hs.438863	NR1H3	Nuclear receptor subfamily 1, group H, member 3
						Hs.386567	GBP2	Guanylate binding protein 2, interferon-inducible
10	Elk-1 (V$ELK1_01) Elk-1	1891	4	0.003677935	0.065622096	Hs.455323	AQP7	Aquaporin 7
						Hs.188591	FLJ22104	Hypothetical protein FLJ22104
						Hs.438863	NR1H3	Nuclear receptor subfamily 1, group H, member 3
						Hs.524418	GPD1	Glycerol-3-phosphate dehydrogenase 1 (soluble)

Rank	Transcription Factor Name (Matrix ID)	Total	Found	P-Value	Q-Value	UniGeneID	Gene Symbol	Gene Name
11	GFI1 (V$GFI1_Q6)	1890	4	0.003670976	0.069136718	Hs.491597	VDAC3	Voltage-dependent anion channel 3
						Hs.188591	FLJ22104	Hypothetical protein FLJ22104
						Hs.438863	NR1H3	Nuclear receptor subfamily 1, group H, member 3
						Hs.386567	GBP2	Guanylate binding protein 2, interferon-inducible
12	Poly A (V$LDSPOLYA_B) Lentiviral Poly A downstream element	1887	4	0.003650155	0.072788393	Hs.310512	CCRL1	Chemokine (C-C motif) receptor-like 1
						Hs.491597	VDAC3	Voltage-dependent anion channel 3
						Hs.386567	GBP2	Guanylate binding protein 2, interferon-inducible
						Hs.524418	GPD1	Glycerol-3-phosphate dehydrogenase 1 (soluble)
13	LXR (V$LXR_Q3)	316	2	0.00536938	0.075842496	Hs.519162	BTG2	BTG family, member 2
						Hs.188591	FLJ22104	Hypothetical protein FLJ22104
14	Pax-4 (V$PAX4_01) Pax-4 binding sites	1885	4	0.00363632	0.077044531	Hs.134974	GAP43	Growth associated protein 43
						Hs.534074	NFATC1	Nuclear factor of activated T-cells, cytoplasmic, calcineurin-dependent 1
						Hs.439312	PLTP	Phospholipid transfer protein
						Hs.405662	CRABP2	Cellular retinoic acid binding protein 2
15	PPAR (V$PPARG_03) PPAR (peroxisome proliferator-activated receptor gamma)	1880	4	0.00360189	0.081402706	Hs.455323	AQP7	Aquaporin 7
						Hs.188591	FLJ22104	Hypothetical protein FLJ22104
						Hs.438863	NR1H3	Nuclear receptor subfamily 1, group H, member 3
						Hs.405662	CRABP2	Cellular retinoic acid binding protein 2
16	v-Maf (V$VMAF_01) v-Maf	1873	4	0.003554065	0.086059141	Hs.439312	PLTP	Phospholipid transfer protein
						Hs.438863	NR1H3	Nuclear receptor subfamily 1, group H, member 3
						Hs.436186	ARTS-1	Type 1 tumor necrosis factor receptor shedding aminopeptidase regulator
						Hs.405662	CRABP2	Cellular retinoic acid binding protein 2
17	E2F-1 (V$E2F1_Q4) E2F-1	1856	4	0.003439742	0.089697888	Hs.519162	BTG2	BTG family, member 2
						Hs.310512	CCRL1	Chemokine (C-C motif) receptor-like 1
						Hs.534074	NFATC1	Nuclear factor of activated T-cells, cytoplasmic, calcineurin-dependent 1
						Hs.524418	GPD1	Glycerol-3-phosphate dehydrogenase 1 (soluble)
18	E2 (V$E2_Q6) papilloma virus regulator E2	1685	4	0.002427171	0.091423435	Hs.519162	BTG2	BTG family, member 2
						Hs.439312	PLTP	Phospholipid transfer protein
						Hs.436186	ARTS-1	Type 1 tumor necrosis factor receptor shedding aminopeptidase regulator
						Hs.405662	CRABP2	Cellular retinoic acid binding protein 2
19	PPAR direct repeat 1 (V$PPAR_DR1_Q2)	1842	4	0.003347517	0.094567352	Hs.455323	AQP7	Aquaporin 7
						Hs.188591	FLJ22104	Hypothetical protein FLJ22104
						Hs.438863	NR1H3	Nuclear receptor subfamily 1, group H, member 3
						Hs.405662	CRABP2	Cellular retinoic acid binding protein 2
20	E2 (V$E2_Q6_01)	768	3	0.002280735	0.09664616	Hs.519162	BTG2	BTG family, member 2
						Hs.439312	PLTP	Phospholipid transfer protein
						Hs.405662	CRABP2	Cellular retinoic acid binding protein 2

CRSD: a comprehensive web server for composite regulatory signature discovery
P value threshold: 0.05
N value: 54576
There are 26 accession numbers in the request (transfer to UniGene ID):

Hs.525324(NM_001262) Hs.439312(NM_006227) Hs.201253(D43948) Hs.438863(NM_005693) Hs.188591(NM_022918) Hs.519162(NM_006763) Hs.524418(NM_005276) Hs.515465(NM_000041) Hs.310512(NM_016557) Hs.386567(NM_004120) Hs.405662(NM_001878) Hs.59729(NM_020163) Hs.491597(NM_005662) Hs.134974(NM_002045) Hs.436186(NM_016442) Hs.455323(NM_001170) Hs.26516(NM_018154) Hs.534074(NM_006162) Hs.446685(NM_006821)
There were 19 unique genes found.
