# Supplementary material for: Comparative transcriptomics of human multipotent stem cells during adipogenesis and osteoblastogenesis
Source: BMC Genomics. 2008 Jul 17;9:340. doi: 10.1186/1471-2164-9-340 (PMC2492879; doi:10.1186/1471-2164-9-340)
Supplement: Additional file 7 — Significantly over-represented transcription factor binding sites. Significantly over-represented transcription factor binding sites of 39 genes with a specific profile for the osteogenic commitment. [file 1471-2164-9-340-S7.pdf]

Additional file 7

Significantly over-represented transcription factor binding sites (TFBS) of 39 genes with a specific profile for the osteogenic commitment.

Rank	Transcription Factor Name (Matrix ID)	Total	Found	P-Value	Q-Value	UniGeneID	Gene Symbol	Gene Name
1	GATA-1 (V$GATA1_02) GATA-binding factor 1	1908	5	0.002213661	0.049392313	Hs.506276	ATP2B1	ATPase, Ca++ transporting, plasma membrane 1
						Hs.553496	PGM3	Phosphoglucomutase 3
						Hs.1908	PRG1	Proteoglycan 1, secretory granule
						Hs.154073	SLC35B1	Solute carrier family 35, member B1
						Hs.494192	OSTF1	Osteoclast stimulating factor 1
2	CBF (core binding factor) (V$CBF_01) CBF sites selected in the presence of Ets-1	1897	5	0.002158621	0.051375171	Hs.435215	VEGFC	Vascular endothelial growth factor C
						Hs.291196	ATP1B1	ATPase, Na+/K+ transporting, beta 1 polypeptide
						Hs.144936	IMP-1	IGF-II mRNA-binding protein 1
						Hs.553496	PGM3	Phosphoglucomutase 3
						Hs.134830	COL8A1	Collagen, type VIII, alpha 1
3	Retroviral Poly A (V$POLY_C) Retroviral Poly A signal	546	3	0.00244714	0.051389931	Hs.209561	KIAA1715	KIAA1715
						Hs.435850	LYPLA1	Lysophospholipase I
						Hs.443852	ZDHHC2	Zinc finger, DHHC-type containing 2
4	AP-4 (V$AP4_Q6_01)	1896	5	0.002153667	0.054918519	Hs.553496	PGM3	Phosphoglucomutase 3
						Hs.154073	SLC35B1	Solute carrier family 35, member B1
						Hs.494192	OSTF1	Osteoclast stimulating factor 1
						Hs.227067	ATAD3A	ATPase family, AAA domain containing 3A
						Hs.126137	BACH	Acyl-CoA thioesterase 7
5	SREBP (V$SREBP_Q3)	1895	5	0.002148723	0.059007228	Hs.506276	ATP2B1	ATPase, Ca++ transporting, plasma membrane 1
						Hs.144936	IMP-1	IGF-II mRNA-binding protein 1
						Hs.494192	OSTF1	Osteoclast stimulating factor 1
						Hs.227067	ATAD3A	ATPase family, AAA domain containing 3A
						Hs.344400	MPHOSPH6	M-phase phosphoprotein 6
6	AP-1 (V$AP1_Q2) activator protein 1	1888	5	0.002114343	0.06290169	Hs.529782	VCP	Valosin-containing protein
						Hs.443852	ZDHHC2	Zinc finger, DHHC-type containing 2
						Hs.227067	ATAD3A	ATPase family, AAA domain containing 3A
						Hs.126137	BACH	Acyl-CoA thioesterase 7
						Hs.344400	MPHOSPH6	M-phase phosphoprotein 6
7	DEAF1 (V$DEAF1_02)	614	3	0.003403234	0.067497482	Hs.475103	NUP50	Nucleoporin 50kDa
						Hs.553496	PGM3	Phosphoglucomutase 3
						Hs.227067	ATAD3A	ATPase family, AAA domain containing 3A
8	XFD-3 (V$XFD3_01) Xenopus fork head domain factor 3	1885	5	0.002099733	0.068145878	Hs.435215	VEGFC	Vascular endothelial growth factor C
						Hs.369438	ETS1	V-ets erythroblastosis virus E26 oncogene homolog 1 (avian)
						Hs.553496	PGM3	Phosphoglucomutase 3
						Hs.134830	COL8A1	Collagen, type VIII, alpha 1
						Hs.494192	OSTF1	Osteoclast stimulating factor 1
9	TFE (V$TFE_Q6)	1878	5	0.002065933	0.073753819	Hs.529782	VCP	Valosin-containing protein
						Hs.506276	ATP2B1	ATPase, Ca++ transporting, plasma membrane 1
						Hs.477155	ATP6V1A	ATPase, H+ transporting, lysosomal 70kDa, V1 subunit A
						Hs.494192	OSTF1	Osteoclast stimulating factor 1
						Hs.344400	MPHOSPH6	M-phase phosphoprotein 6
10	Poly A downstream element (V$PADS_C) Retroviral Poly A downstream element	1876	5	0.00205635	0.081568565	Hs.369438	ETS1	V-ets erythroblastosis virus E26 oncogene homolog 1 (avian)
						Hs.477155	ATP6V1A	ATPase, H+ transporting, lysosomal 70kDa, V1 subunit A
						Hs.446554	RAD51	RAD51 homolog (RecA homolog, E. coli) (S. cerevisiae)
						Hs.227067	ATAD3A	ATPase family, AAA domain containing 3A
						Hs.344400	MPHOSPH6	M-phase phosphoprotein 6
11	CBF (core binding factor) (V$CBF_02) CBF sites selected in the absence of Ets-1	1876	5	0.00205635	0.091764636	Hs.435215	VEGFC	Vascular endothelial growth factor C
						Hs.291196	ATP1B1	ATPase, Na+/K+ transporting, beta 1 polypeptide
						Hs.144936	IMP-1	IGF-II mRNA-binding protein 1
						Hs.553496	PGM3	Phosphoglucomutase 3
						Hs.134830	COL8A1	Collagen, type VIII, alpha 1

CRSD: a comprehensive web server for composite regulatory signature discovery
P value threshold: 0.05
N value: 54576
There are 39 accession numbers in your request (transfer to UniGene ID):

Hs.534612(BC007382) Hs.144936(AF117106) Hs.494192(NM_012383) Hs.446554(NM_002875) Hs.435850(NM_006330) Hs.553496(NM_015599) Hs.21432(NM_017514) Hs.2820(NM_000916) Hs.134830(NM_020351) Hs.471873(NM_012145) Hs.126137(NM_007274) Hs.519445(NM_005654) Hs.475103(NM_007172) Hs.443852(NM_016353) Hs.154073(NM_005827) Hs.435215(NM_005429) Hs.1908(NM_002727) Hs.506276(NM_001682) Hs.209561(AB051502) Hs.435981(NM_001983) Hs.344400(NM_005792) Hs.227067(NM_018188) Hs.477155(NM_001690) Hs.529782(NM_007126) Hs.443021(NM_003532) Hs.291196(NM_001677) Hs.369438(NM_005238)
There were 27 unique genes found.
